# Polyphosphate Accumulation Is Determined by Zinc and Inositol in *Saccharomyces cerevisiae*

**DOI:** 10.1002/yea.70006

**Published:** 2025-10-25

**Authors:** Alexander Deitert, Makarius Baier, Roy Eerlings, Jana Fees, Ailín Österlein Kück, Julia Repin, Philipp Demling, Lars M. Blank

**Affiliations:** ^1^ Institute of Applied Microbiology – iAMB, Aachen Biology and Biotechnology – ABBt, Worringer Weg 1 RWTH Aachen University Aachen Germany

**Keywords:** inositol, phosphate starvation, polyphosphate, *Saccharomyces cerevisiae*, yeast, zinc

## Abstract

Polyphosphate (polyP) is an intriguing polymer with diverse biological and industrial applications. Chemical polyP production is energy‐intensive and limited in chain length at large‐scale production. Alternatively, biological production offers a sustainable solution. Recent research endeavors highlighted *Saccharomyces cerevisiae* as a promising organism for polyP hyperaccumulation, achieving up to 28% (w/w) polyP (as KPO_3_). P_i_ starvation and P_i_ feeding are essential for this hyperaccumulation phenotype. Prior research demonstrated that trace elements and vitamins increase polyP production in *S. cerevisiae* when added to the cultivation medium during P_i_ starvation. However, the role of trace elements and vitamins in enhancing polyP accumulation remained unclear. This study identified inositol and zinc to drive polyP accumulation across various laboratory and industrial *S. cerevisiae* strains. Moreover, these components influence the energy metabolism of yeasts. Our findings suggest that zinc boosts the phosphate‐responsive signal transduction (PHO) pathway during P_i_ starvation. The influence of inositol on polyP hyperaccumulation remains elusive, as it does not influence the PHO pathway directly. These findings add to the ever‐growing understanding of polyP metabolism in *S. cerevisiae* and provide further targets for optimizing biological polyP production.

## Summary

- Zinc and inositol both increase polyP hyperaccumulation and energy metabolism in *Saccharomyces cerevisiae*.
- Effect of zinc and inositol confirmed in laboratory and industrial yeast strains.
- Zinc directly influences polyP accumulation through PHO pathway activation.
- The role of inositol in enhanced polyP hyperaccumulation remains elusive.

## Graphical abstract

Zinc and inositol supplementation during phosphate starvation significantly enhances final polyphosphate levels in *S. cerevisiae*.

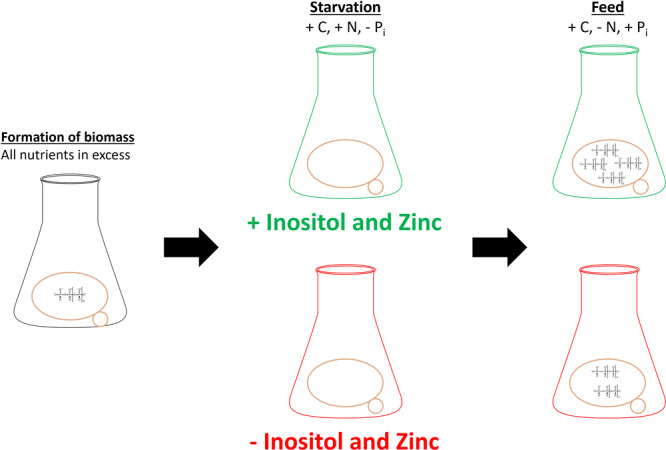

## 1 Introduction

Inorganic polyphosphate (polyP) is the linear polymer of phosphate (P_i_/PO_4_^3‐^) connected by phosphoanhydride bonds and consisting of two to several thousand P_i_ subunits (Brown and Kornberg 2008; Rao et al. 2009). PolyP plays a central role in various cellular processes, including energy‐ and phosphate storage, cellular stress response, and membrane transport (Kulaev et al. 2004). In humans and animals, it is also involved in biomineralization and blood coagulation (Kulakovskaya et al. 2012; Kullik et al. 2024). Its unique and diverse physicochemical properties, partially dependent on the chain length, deem polyP of interest for industrial purposes (Kulakovskaya et al. 2012; Rashchi and Finch 2000). Biodegradability, non‐flammability, and non‐toxicity are properties independent of the polyP chain length, suiting it in fertilizers, flame retardants, and food additives (Demling et al. 2024; Kulakovskaya et al. 2012). Other properties, including pH‐buffering capacity, are more pronounced in short‐chain polyPs. In addition, polyP_2_ and polyP_3_ play a unique role in increasing the water‐holding capacity of meat after rigor mortis (Shen and Swartz 2010). Properties such as cation‐complexing ability and bacteriostatic activity are more pronounced in long‐chain polyPs, which are of interest for the food industry (EFSA Panel on Food Additives and Flavourings (FAF) et al. 2019; Kulakovskaya et al. 2012; Lorencová et al. 2012). PolyP is produced chemically from phosphoric acid (obtained from phosphate rock) by a condensation reaction at high temperatures (van Wazer 1958). Besides being highly energy‐intensive and based on fossil resources, production is limited to manufacturable polyP chain lengths of 2 ‐ 40 P_i_ subunits at large scale (Rashchi and Finch 2000). Projected environmental benefits, including less energy consumption at benign process conditions and the potential to recycle different phosphate sources, thrived the search for alternative, biological polyP production processes. It has been known since the 1960s that organisms such as algae and yeast selectively take up P_i_ and polymerize it intracellularly to polyP under certain conditions (Liss and Langen 1962; Plouviez et al. 2021). This cellular process is referred to as polyP hyperaccumulation. PolyP hyperaccumulation has been primarily studied from a genetic and physiological perspective in microbes (Austin and Mayer 2020; Gerasimaitė et al. 2014). Despite polyP's industrial relevance, few attempts have been made to produce polyP‐rich biomass or pure biotechnologically synthesized (bio‐)polyP (Christ and Blank 2019; Christ et al. 2020).

Previous studies demonstrated that *Saccharomyces cerevisiae* can be an efficient and robust polyP production host. Moreover, *S. cerevisiae* is generally considered safe (GRAS), it is easy to cultivate, and yeast extract production is industrially established (Parapouli et al. 2020; Tao et al. 2023; van der Hoek et al. 2019). Christ and Blank (2019) reported a cultivation strategy for polyP hyperaccumulation involving P_i_ starvation and subsequent P_i_ feeding, in which baker's yeast produces up to 28% (w/w) polyP (as KPO_3_) per cell dry weight. The robustness of the strategy was demonstrated using industrial waste streams or plant residues as phosphate sources for polyP production (Fees et al. 2023; Herrmann et al. 2023). Prior research highlighted that trace elements and vitamins drastically increase polyP production in *S. cerevisiae* when added to the cultivation medium during the starvation phase of the hyperaccumulation protocol (Christ and Blank 2019). However, the observed effects were not pinpointed to individual components of the cultivation medium.

In this study, we identify the individual components of the trace elements and vitamins in the cultivation medium responsible for elevated polyP production in the hyperaccumulation protocol. After identification, the effect of the components was confirmed for industrial and laboratory yeast strains. In addition, initial attempts were made to determine their mode of action in the polyP metabolism. Thereby, this study improves the understanding of polyP hyperaccumulation in *S. cerevisiae* and provides a starting point for identifying mechanisms involved in polyP accumulation.

## 2 Materials and Methods

### 2.1 Strains

The industrial strains *S. cerevisiae* VH2.200 and *S. cerevisiae* Ethanol Red were used in this study (hereinafter referred to as VH2.200 and Ethanol Red, respectively). VH2.200 was provided by the VH Berlin e.V. (Research Institute for Baker's Yeast, Berlin, Germany) and Ethanol Red was provided by Leaf (Lesaffre, Marcq‐en‐Baroeul, France). Moreover, the laboratory strain *S. cerevisiae* CEN.PK 113‐7D (hereinafter referred to as CEN.PK WT) and the engineered strain *S. cerevisiae* CEN.PK 113‐7D pPr_Pho5_‐sfGFP were studied. *S. cerevisiae* CEN.PK 113‐7D pPr_Pho5_‐sfGFP was generated by introducing the plasmid pPr_Pho5_‐sfGFP into *S. cerevisiae* CEN.PK 113‐7D (hereinafter referred to as CEN.PK pPr_Pho5_‐sfGFP). The gene encoding for the fluorescent reporter protein sfGFP was expressed under the control of the Pho5 promoter to investigate the phosphate‐responsive signal transduction (PHO) pathway activation (Supplementary Figure 4) (Chabert et al. 2023).

### 2.2 Cultivation Conditions for Producing PolyP‐Rich Cells

The hyperaccumulation protocol established by Christ and Blank (2019) was applied to produce polyP‐rich yeasts. In brief, the protocol first involved a *S. cerevisiae* preculture by inoculating 10 mL SD‐medium (6.80 g/L yeast nitrogen base without amino acids, 20.00 g/L glucose, pH 5.4) with single colonies from a YEP‐agar plate (30°C, 200 rpm, 100 mL flask, 24 h). 1% (v/v) inoculum from the preculture was then used for cell mass production in 50 mL SD‐medium (30°C, 200 rpm, 500 mL flask, 24 h). Subsequently, the cell mass was harvested through centrifugation (5000 g, 5 min), washed with deionized water, air‐dried for 5 min on P_i_‐free filter paper, and stored at 4°C (maximum of 2 weeks). 2.5 g/L *S. cerevisiae* cell wet weight (CWW) was then cultivated in 50 mL starvation medium (23.96 g/L glucose, 1.32 g/L (NH_4_)_2_SO_4_, 0.99 g/L KCl, 0.58 g/L CaCl_2_ · 2 H_2_O, 2.02 g/L disodium succinate; trace elements (TE): 15.0 mg/L Na_2_EDTA, 4.5 mg/L ZnSO_4_ · 7 H_2_O, 1.0 mg/L MnCl_2_ · 2 H_2_O, 0.3 mg/L CoCl_2_ · 6 H_2_O, 0.3 mg/L CuSO_4_ · 5 H_2_O, 0.4 mg/L Na_2_MoO_4_ · 2 H_2_O, 0.1 mg/L KI, 4.5 mg/L CaCl_2_ · 2 H_2_O, 3.0 mg/L FeSO_4_ · 7 H_2_O, 1 mg/L H_3_BO_3_; trace vitamins (TV): 0.05 mg/L biotin, 1.0 mg/L nicotinic acid, 1.0 mg/L Ca‐d‐pantothenate, 0.2 mg/L p‐aminobenzoic acid, 1.0 mg/L pyridoxal hydrochloride, 1.0 mg/L thiamine hydrochloride, 25.0 mg/L myo‐inositol, pH 5.0) at 30°C and 200 rpm in a 500 mL flask for 6 h. At the end of each starvation phase, the cells were harvested (5000 g, 5 min), washed once with deionized water, and dried on filter paper (5 min). This was followed by storage of the cells at 4°C until the start of the feeding phase (17 h). The P_i_‐starved *S. cerevisiae* cells were then transferred to 10 mL feeding medium (component A (1.11x concentrated): 49.99 g/L glucose, 9.06 g/L KH_2_PO_4_, pH 6.0; component B (10x concentrated): 40.66 g/L MgCl_2_; unified to a ratio of 9:1 component A: component B before inoculation). A maximal concentration of 30 g/L CWW was applied and cultivated (30°C, 200 rpm, 100 mL flask, 2.5 h). Afterward, P_i_‐fed *S. cerevisiae* cells were harvested by centrifugation (5000 g, 5 min), washed twice with deionized water, dried on filter paper (5 min), and stored at −20°C until polyP analysis (Christ and Blank 2018a, 2018b).

### 2.3 Modification of Trace Elements and Vitamins in the Starvation Medium

Verduyn trace elements and vitamins were supplemented to the starvation medium according to Christ and Blank (2019). The concentrations refer to the mineral salts medium designed by Verduyn et al. (1992). To investigate the effect of individual trace elements and vitamins on polyP production, *S. cerevisiae* was cultivated in modified versions of the starvation medium. For this, trace element and vitamin solutions containing only a single component were prepared (Table S1 and S2). To narrow down specific trace elements influencing the polyP accumulation, all trace elements were clustered into three different groups (Table 1).

**Table 1:** Clusters of trace elements applied for screening. Trace elements were randomly assigned to the respective groups.

Group 1	Group 2	Group 3
Na_2_EDTA	CoCl_2_ · 6 H_2_O	FeSO_4_ · 7 H_2_O
ZnSO_4_ · 7 H_2_O	CuSO_4_ · 5 H_2_O	H_3_BO_3_
MnCl_2_ · 2 H_2_O	Na_2_MoO_4_ · 2 H_2_O	CaCl_2_ · 2 H_2_O
	KI	

In a series of cultivations, one group of trace elements each was omitted. PolyP production with *S. cerevisiae* was then compared to a fully supplemented starvation medium (positive control, PC) and starvation medium without any trace elements (NE_TE_). Next, if the omission of a specific trace element group led to reduced polyP levels, *S. cerevisiae* was cultivated in a starvation medium lacking each component from the respective group individually. The vitamins were examined by omitting each individually from the starvation medium in a series of cultivations. The results were then compared to the PC and starvation medium without vitamins (NE_TV_). Finally, polyP hyperaccumulation was performed in a starvation medium containing only the trace elements and vitamins identified to be beneficial for polyP production. Results were then compared to the PC and starvation medium without trace elements and vitamins (NE_TE+TV_).

### 2.4 Determination of Oxygen and Carbon Dioxide Transfer Rates

The Transfer‐Rate Online Measurement (TOM) device (Adolf Kuhner AG, Birsfelden, Switzerland) was applied to monitor the respiratory activity of *S. cerevisiae* cultures during the starvation phase by noninvasive online determination of the oxygen transfer rate (OTR) and carbon dioxide transfer rate (CTR) in up to eight individual shake flasks. OTR and CTR were calculated according to Anderlei et al. (2004). Starvation experiments were conducted in 50 mL medium according to the abovementioned conditions (30°C, 200 rpm, 500 mL flask, 6 h). The OTR and CTR measurements were performed in 20‐min cycles. These are divided into a 5‐min measuring phase, a 1.17‐min flushing phase, and a 13.83‐min aeration phase.

### 2.5 BioLector Cultivation

Starvation experiments with CEN.PK pPr_Pho5_‐sfGFP were performed in the BioLector I (Beckman Coulter GmbH, Baesweiler, Germany) in 48‐well flower plates (M2P‐MTP‐48‐B, Beckman Coulter GmbH, Baesweiler, Germany) to monitor GFP expression and growth online. Cultivations in the BioLector were conducted at 30°C, 1,200 rpm, and 85% humidity. The filling volume in the plates was 1.2 mL. 0.2 mg/L geneticin (G418) was added to all cultivations with CEN.PK pPr_Pho5_‐sfGFP to ensure plasmid retention. All other parameters are based on the above‐mentioned conditions of the starvation phase. GFP (gain: 90, λ_ex_: 488 nm, λ_em_: 520 nm) and scattered light (gain: 50, λ_ex_: 620 nm, λ_em_: 620 nm) were measured with two internal filter modules. The OD‐corrected GFP signal was calculated by correlating GFP to the scattered light signal.

### 2.6 Fermentation Broth Analysis

Glucose and ethanol in the starvation medium were determined using a high‐performance liquid chromatography (HPLC‐UV‐RI) system (DIONEX UltiMate 3000 consisting of the autosampler WPS‐3000 (RS), the column oven TCC‐3000 (RS) and the pump module LPG‐3400SD, all Thermo Fisher Scientific, Germany) with an ISERA Metab AAC 300 × 7.8 mm separation column (particle size: 10 μm, ISERA GmbH, Düren, Germany). Noncultivated starvation medium and cultivation supernatant after starvation were measured. Samples were diluted 1:2 with deionized water and filtered (Berrytec syringe filters, pore size: 0.22 μm, *d* = 13 mm, Berrytec GmbH, Harthausen, Germany) before the measurement. The column temperature was set to 40°C, and the run time of the method was 25 min per sample. For the isocratic method, a mobile phase of 5 mM H_2_SO_4_ was pumped at a constant flow rate of 0.6 mL/min. The injection volume of the samples was 5 μL. The SHODEX RI‐101 refractive index (RI) detector (Showa Denko Europe GmbH, Germany) was used for glucose and ethanol detection.

### 2.7 Enzymatic PolyP Analytics

PolyP was extracted from polyP‐enriched *S. cerevisiae* via phenol/chloroform extraction (Christ and Blank 2018a). The Phosfinity kit from Aminoverse B.V. (Nutz, Netherlands) was used according to the supplied protocol to determine the P_i_ and polyP concentration (Christ and Blank 2018b). PolyP levels for the initial screening of relevant trace elements and vitamins are expressed in relation to the PC. Otherwise, polyP values in this study refer to cellular polyP contents (as KPO_3_) in percent in cell dry weight [%KPO_3_ (w/w)]. These polyP values can be converted to the percentage of phosphorus in cell dry weight [%P (w/w)] by applying a factor of 0.26.

### 2.8 Statistical Analysis

One‐way ANOVA via the GraphPad Prism software version 13.02 (GraphPad Software Inc., California, USA) was applied to test the experimental results for significance (*p ≤ 0.05, **p ≤ 0.01, ***p ≤ 0.001, ****p ≤ 0.0001).

## 3 Results

With the overall goal of characterizing polyP production by *S. cerevisiae* in our previously published polyP hyperaccumulation protocol, we investigated the role of the individual trace elements and vitamins within the starvation phase, thereby saving resources.

### 3.1 Zinc Is the Sole Beneficial Trace Element for PolyP Production

**Figure 1:**
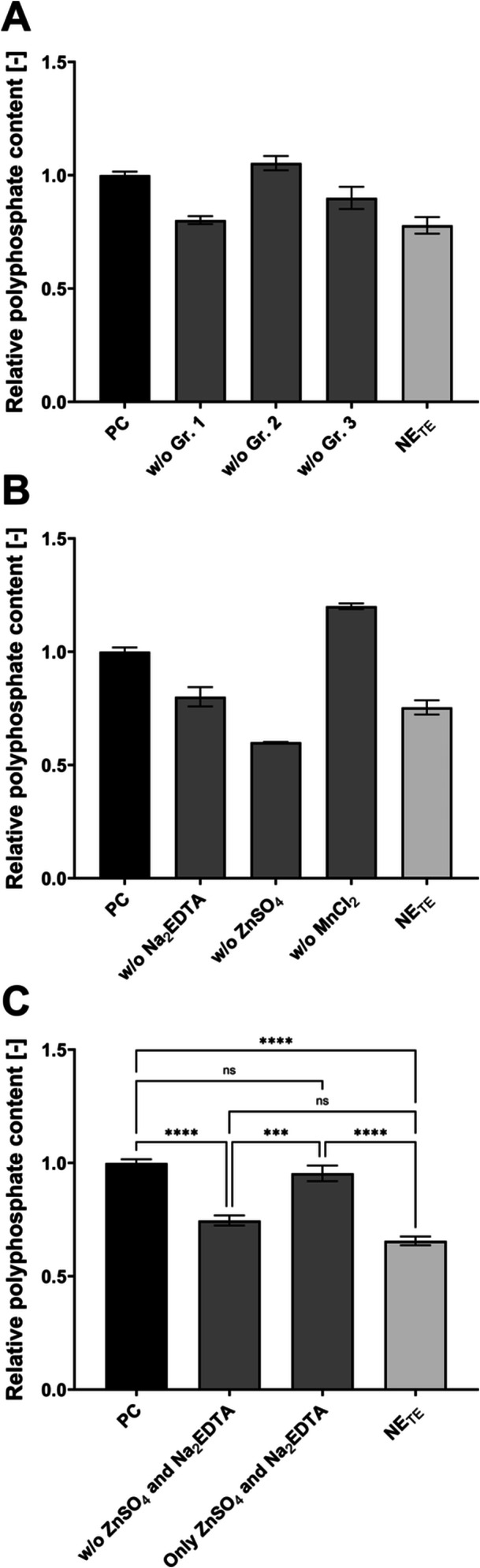
Influence of trace elements on polyP hyperaccumulation of VH2.200. Cultivation in starvation medium with different combinations of trace elements. PC (all trace elements) and NE_TE_ (w/o any trace elements). (A) Omission of groups of trace elements. PC, w/o Group 1 (Na_2_EDTA, ZnSO_4,_ and MnCl_2_), w/o Group 2 (CoCl_2_, CuSO_4_, Na_2_MoO_4,_ and KI), w/o Group 3 (FeSO_4,_ H_3_BO_3_, and CaCl_2_), and NE_TE_ (*n* = 2); (B) Individual omission of trace elements of Group 1. PC, w/o Na_2_EDTA, w/o ZnSO_4_, w/o MnCl_2_, and NE_TE_ (*n* = 2); (C) Combined effect of added Na_2_EDTA and ZnSO_4_. PC (*n* = 6), w/o Na_2_EDTA and ZnSO_4_ (*n* = 4), only with Na_2_EDTA and ZnSO_4_ (*n* = 4), and NE_TE_ (*n* = 6). Data are presented as means ± SEM. Significant differences marked by asterisks (*p ≤ 0.05, **p ≤ 0.01, ***p ≤ 0.001, ****p ≤ 0.0001; One‐way ANOVA).

The sub‐groups of the original trace element composition (Table 1) were used in a series of cultivations to narrow down the trace elements in the starvation phase responsible for elevated polyP production (Figure 1A).

The omission of Group 1 led to a relative polyP content of 0.80 ± 0.02, of Group 2 to 1.05 ± 0.04, and of Group 3 to 0.90 ± 0.07 compared to the fully supplemented positive control (PC). The omission of all trace elements (NE_TE_) resulted in a relative polyP content of 0.78 ± 0.05. Only the omission of Group 1 led to a significant polyP decrease. Therefore, the contribution of the individual trace elements of Group 1 to polyP hyperaccumulation was examined. Although the omission of Group 3 showed a nonsignificant 10% decrease in polyP content compared to the PC (1.00 ± 0.02), the individual examination of Group 3 components did not influence polyP hyperaccumulation (Figure S1). The cultivations lacking individual components of Group 1 (Figure 1B) resulted in a relative polyP content of 0.60 ± 0 and 0.80 ± 0.06 without (w/o) ZnSO_4_ and Na_2_EDTA, respectively. On the contrary, cultivations without MnCl_2_ resulted in a relative polyP content of 1.20 ± 0.02. The omission of all trace elements (NE_TE_) resulted in a relative polyP content of 0.75 ± 0.04. Therefore, ZnSO_4_ and Na_2_EDTA were selected as beneficial trace elements for polyP production. As the final adjusted starvation medium contains only relevant trace elements if present, MnCl_2_ is omitted. The observed positive impact of its absence on polyP production is thereby covered. Thus, the effect of absent MnCl_2_ was not investigated further. The role of Na_2_EDTA and ZnSO_4_ was verified by adding only these two components or all other trace elements except Na_2_EDTA and ZnSO_4_ (Figure 1C). Adding only Na_2_EDTA and ZnSO_4_ resulted in a relative polyP content of 0.95 ± 0.03, representing a no significant difference to the PC. NE_TE_ resulted in a lower relative polyP content of 0.66 ± 0.02 compared to these approaches (*p* ≤ 0.0001). Similarly, when adding all trace elements in the starvation except for Na_2_EDTA and ZnSO_4_, the relative polyP content was reduced (0.75 ± 0.02). Hence, the relative content of the PC was significantly higher (*p* ≤ 0.0001), but no significant difference to the NE_TE_ is detectable for this approach. This confirmed the impact of Na_2_EDTA and ZnSO_4_ on polyP hyperaccumulation, while all other trace elements added in the original protocol can be omitted.

### 3.2 Inositol Is the Sole Beneficial Compound in the Vitamin Mixture

**Figure 2:**
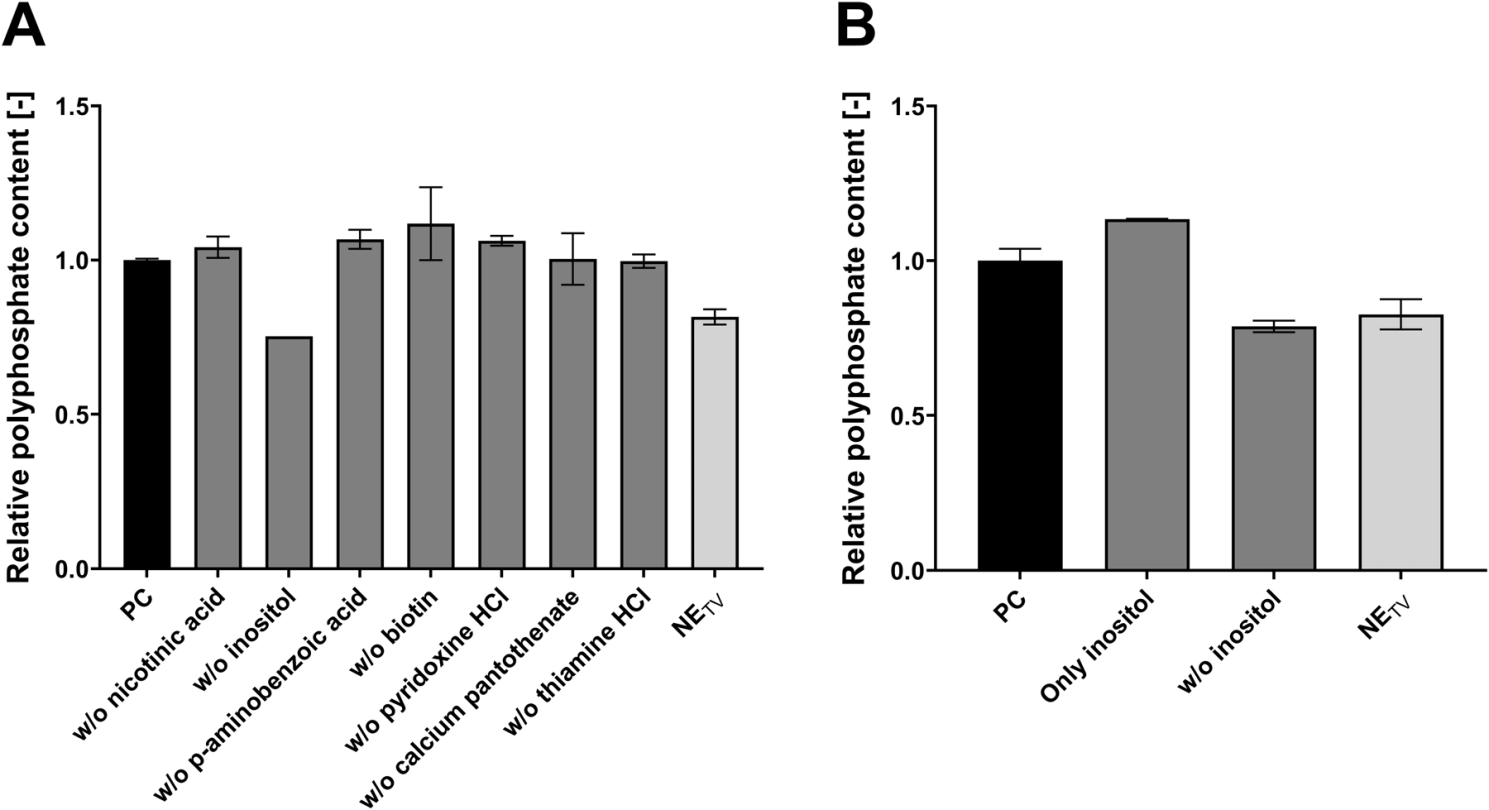
Influence of individual vitamins on polyP hyperaccumulation of VH2.200. Cultivation in starvation medium with different combinations of vitamins. PC (all vitamins) and NE_TV_ (w/o any vitamins). (A) w/o nicotinic acid, w/o inositol, w/o p‐aminobenzoic acid, w/o biotin, w/o pyridoxine hydrochloride, w/o calcium pantothenate, and w/o thiamine hydrochloride; (B) Only with and w/o inositol. All cultivations were performed in duplicates (*n* = 2). Data are presented as means ± SEM.

The vitamin mixture, containing besides bona fide vitamins compounds such as inositol, was individually omitted to determine the components in the starvation phase responsible for elevated polyP production (Figure 2A). The relative polyP content of modified media without nicotinic acid (1.04 ± 0.03), without p‐aminobenzoic acid (1.07 ± 0.03), without biotin (1.12 ± 0.12), without pyridoxine HCl (1.06 ± 0.02), without calcium pantothenate (1.00 ± 0.08), and without thiamine HCl (1.00 ± 0.02) revealed no substantial difference compared to the PC and were not considered for further analysis. In contrast, the modified medium without inositol exhibited a relative polyP level of 0.75 ± 0. This was comparable to the level of 0.82 ± 0.02 when omitting all vitamins (NE_TV_). As a result, inositol was identified as the only beneficial compound in the vitamin mixture for polyP production.

Hyperaccumulation with a cultivation step in modified starvation medium containing only inositol and a second medium containing all vitamins except inositol confirmed its impact on polyP accumulation (Figure 2B). The medium with inositol exhibited a relative polyP content of 1.13 ± 0. Just omitting inositol resulted in a relative content of 0.79 ± 0.03. This is similar to NE_TV_ (0.83 ± 0.07). These results confirm the impact of inositol on polyP accumulation, while all other vitamins added in the original protocol can be neglected.

### 3.3 Inositol and Zinc Are Sufficient for High PolyP Production in *S. cerevisiae* VH2.200

**Figure 3:**
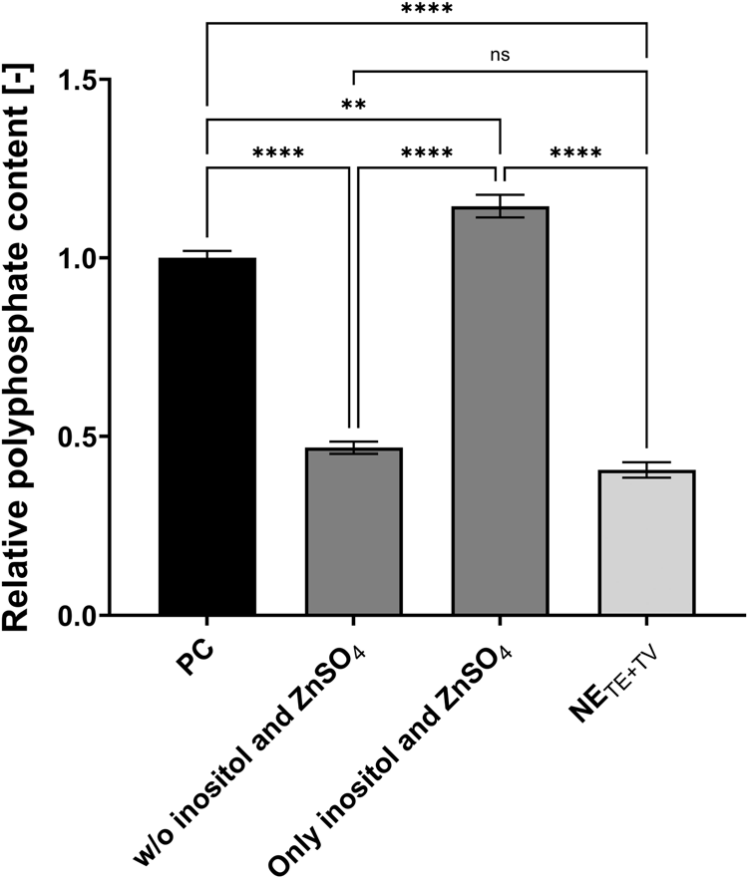
Influence of zinc and inositol on polyP hyperaccumulation of VH2.200**.** Cultivation in modified starvation medium. PC (all vitamins + trace elements, *n* = 6), w/o inositol and ZnSO_4_ (*n* = 4), only with inositol and ZnSO_4_ (*n* = 4), and NE_TE+TV_ (w/o any trace elements and vitamins, *n* = 6). Data are presented as means ± SEM. Significant differences marked by asterisks (*p ≤ 0.05, **p ≤ 0.01, ***p ≤ 0.001, ****p ≤ 0.0001; One‐Way ANOVA).

The identified positive impact of zinc and inositol on polyP hyperaccumulation was combined and tested for synergetic effects (Figure 3).

Modified medium with only inositol and ZnSO_4_ resulted in a relative polyP content of 1.14 ± 0.03, even demonstrating a significant increase compared to the PC (*p* ≤ 0.01). The relative polyP value of the NE_TE+TV_ (0.41 ± 0.02) was significantly lower than the modified medium (*p* ≤ 0.0001). The addition of all other trace elements and vitamins except for inositol and ZnSO_4_ resulted in a significantly lower relative polyP content (0.47 ± 0.02) compared to the PC (1.00 ± 0.02; *p* ≤ 0.0001). A nonsignificant difference compared to the NE_TE+TV_ was also demonstrated. Inositol and ZnSO_4_ independently contributed approximately 34% to the final polyP level (Figure S2). Therefore, both inositol and ZnSO_4_ are beneficial for polyP accumulation. If only the two out of all trace elements and vitamins are present during starvation, high polyP contents can be reached. The final modified medium contained inositol and ZnSO_4_ but no Na_2_EDTA since Na_2_EDTA functions as a chelator (Zhang et al. 2021). Therefore, by omitting the chelator from the starvation medium, the ions of the starvation medium are more accessible to *S. cerevisiae*, further saving resources. Moreover, the number of poorly soluble ions and the number of ions in total have been drastically reduced due to the experiments shown here compared to the original medium composition. The results of Figure 3 confirm no negative impact by omitting Na_2_EDTA from the starvation medium, since even a higher relative polyP content compared to the PC can be obtained with only inositol and ZnSO_4_. These results confirm the individual influence of zinc and inositol to reach high polyP contents with VH2.200. Still, additional yeast strains must be tested to make a more general statement about the impact of zinc and inositol on polyP metabolism.

### 3.4 Validating the Effect of Zinc and Inositol Using Diverse Yeast Strains

**Figure 4:**
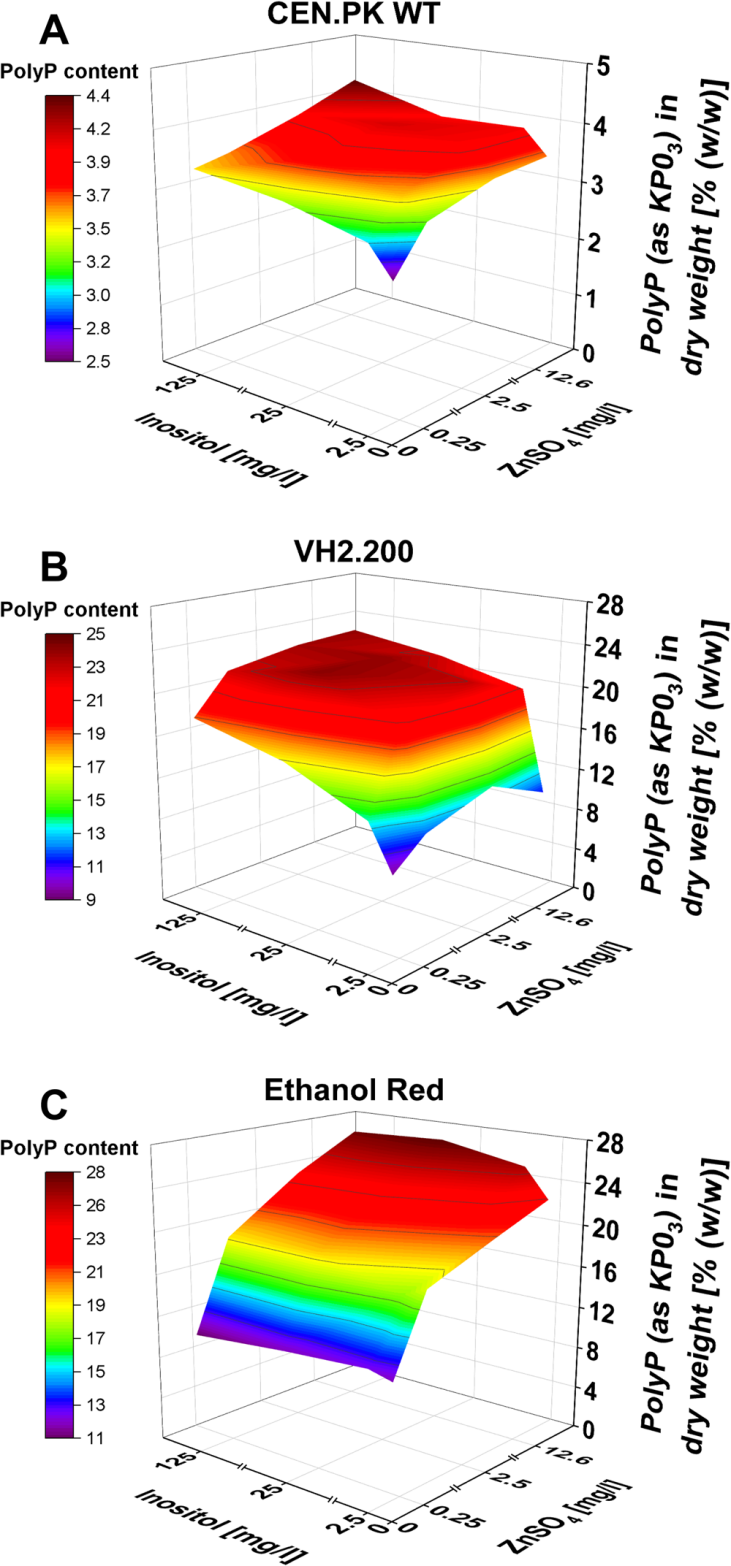
3D‐surface diagrams for the effect of inositol and zinc on polyP hyperaccumulation of diverse yeast strains. PolyP levels with varying inositol (0, 2.5, 25, 125 mg/L) and ZnSO_4_ (0, 0.25, 2.5, 12.6 mg/L) concentrations. (A) *S. cerevisiae* CEN.PK WT; (B) VH2.200; (C) Ethanol Red. The colors of the spanned surface indicate the final polyP content as depicted in the legend: *S. cerevisiae* CEN.PK WT (0%–4.4% (w/w), VH2.200 (0%–25% (w/w)) and Ethanol Red (0%–28% (w/w)). Data are presented as means (*n* = 3).

The combination of various inositol and zinc concentrations in the starvation medium was applied to demonstrate its impact on polyP production for commonly used laboratory (CEN.PK WT) and industrial (VH2.200 and Ethanol Red) *S. cerevisiae* strains (Figure 4). The concentration range was based on previous data by Christ and Blank (2019) as well as preliminary analysis with *S. cerevisiae* VH2.200 (data not shown).

These data demonstrate independently increasing polyP levels with zinc and inositol, as well as strain‐specific concentration responses. In the case of VH2.200 and CEN.PK WT, a slight concentration increase of both substances already elevated the final polyP content substantially. A further increase had relatively small effects. The increase from minimal (0 mg/L inositol, 0 mg/L ZnSO_4_) to optimal concentration level was 2.4‐fold (25 mg/L inositol, 0.25 mg/L ZnSO_4_) and 2.0‐fold (125 mg/L inositol, 12.6 mg/L ZnSO_4_) for VH2.200 and CEN.PK WT, respectively. Interestingly, the polyP level in Ethanol Red is not strongly influenced by the inositol level but more by the zinc concentration. The polyP content of Ethanol Red increased 2.3‐fold from minimal to optimal concentration (25 mg/L inositol, 12.6 mg/L ZnSO_4_). Hence, in cultivations with Ethanol Red inositol can be omitted from the starvation medium to reduce medium costs further. Nevertheless, optimal zinc and inositol concentrations are crucial to achieve maximal polyP production and must be adapted to each yeast strain.

**Figure 5:**
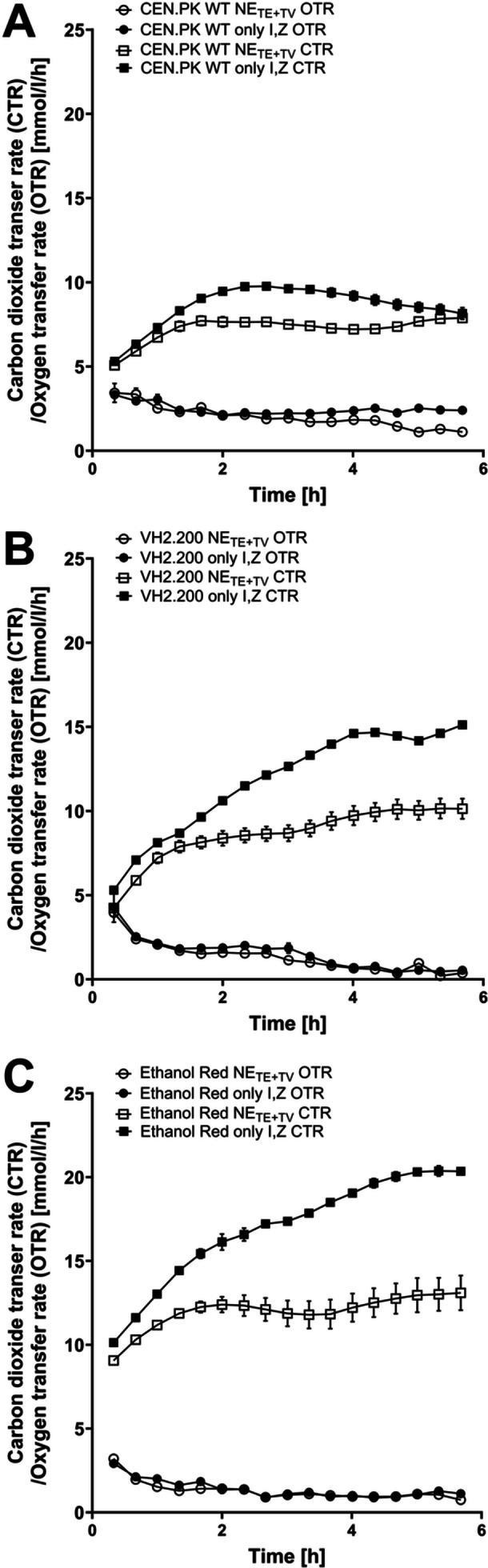
TOM cultivation with various yeast strains in starvation medium without or only with inositol and zinc. Oxygen transfer rate (OTR) and carbon dioxide transfer rate (CTR) for cultivation in starvation medium w/o any trace elements and vitamins (NE_TE+TV_) or only with inositol and ZnSO_4_. (A) *S. cerevisiae* CEN.PK WT (125 mg/L inositol, 12.6 mg/L ZnSO_4_); (B) VH2.200 (25 mg/L inositol, 2.5 mg/L ZnSO_4_); (C) Ethanol Red (25 mg/L inositol, 12.6 mg/L ZnSO_4_). All cultivations were performed in duplicates (*n* = 2). Data are presented as means ± SEM.

TOM cultivations were performed to measure the respiratory activity of the strains during starvation. This confirmed the metabolic effect of zinc and inositol on the yeast strains in the starvation phase (Figure 5). The OTR remained unaffected by these additions, but the CTR was increased in all approaches with zinc and inositol. The CTR increased particularly strongly in cultivations with VH2.200 and Ethanol Red. The increased CTR signals in cultivations with inositol and zinc compared to cultivations without inositol and zinc (NE_TE + TV_) correlated with elevated biomass production, ethanol production, and glucose consumption (Table 2). This all indicated increased fermentative activity.

**Table 2:** Metabolic impact of inositol and zinc on *S. cerevisiae* during phosphate starvation.

		Glucose uptake rate [g/L/h]	Ethanol production rate [g/L/h]	Biomass increase [g/L/h]
VH2.200	NE_TE+TV_	0.94	0.30	0.06
	Only inositol and zinc	1.46	0.53	0.28
CEN.PK WT	NE_TE+TV_	0.93	0.28	0.07
	Only inositol and zinc	1.12	0.34	0.19
Ethanol Red	NE_TE+TV_	1.22	0.43	0.23
	Only inositol and zinc	1.55	0.56	0.34

Moreover, the final polyP content was substantially lower in cultivations without zinc and inositol (Figure S3). Altogether, these measurements confirm the influence of zinc and inositol on polyP hyperaccumulation and the energy metabolism of diverse yeast strains. However, the mode of action of these two components remains unknown.

### 3.5 Identifying Zinc as Activator of the PHO Pathway under P_i_ Starvation Conditions

**Figure 6:**
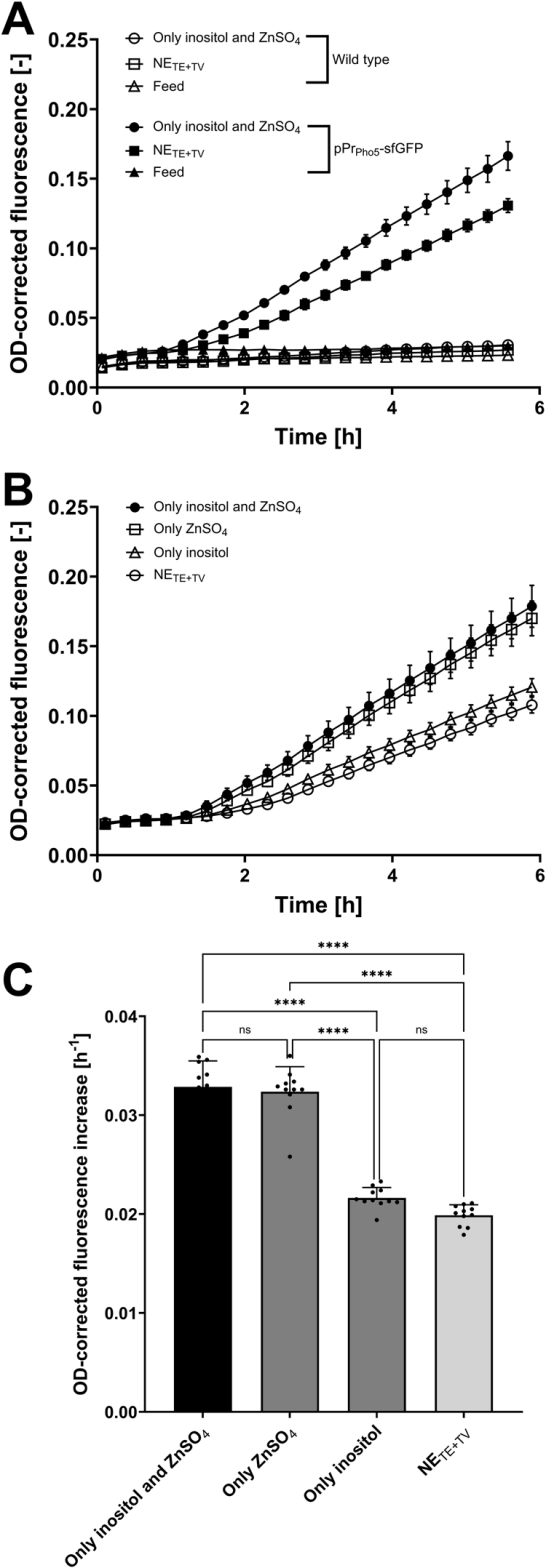
PHO pathway activation by *S. cerevisiae* in the starvation phase of the polyP hyperaccumulation protocol. Assaying PHO pathway activation through live GFP readout during cultivation at different conditions. (A) OD‐corrected fluorescence for CEN.PK WT (*n* = 7) and CEN.PK pPr_Pho5_‐sfGFP (*n* = 8), starvation medium w/o any trace elements and vitamins (NE_TE+TV_), only with inositol and ZnSO_4,_ as well as P_i_‐rich feeding medium; (B) OD‐corrected fluorescence for CEN.PK pPr_Pho5_‐sfGFP (*n* = 11), starvation medium only with inositol and ZnSO_4_, only with ZnSO_4_, only with inositol, and w/o any trace elements and vitamins (NE_TE+TV_); (C) Calculation of rates for OD‐corrected GFP increase between 3 and 6 h of (B). GFP (gain: 90, λ_ex_: 488 nm, λ_em_: 520 nm), biomass (gain: 50, λ_ex_: 620 nm, λ_em_: 620 nm). Data are presented as means ± SD. Significant differences marked by asterisks (*p ≤ 0.05, **p ≤ 0.01, ***p ≤ 0.001, ****p ≤ 0.0001; One‐Way ANOVA).

The plasmid (pPr_Pho5_‐sfGFP) was constructed similar to the published plasmid of Chabert et al. (2023) and applied to determine the mechanism of action of inositol and zinc in polyP synthesis/polyP signaling (Figure S4). The plasmid contains sfGFP, whose expression is controlled by the Pho5 promoter that is regulated by the PHO pathway. The PHO genes guarantee P_i_ homeostasis, specifically at P_i_ starvation conditions (Austin and Mayer 2020). Thereby, the starvation status of the cells can be monitored during cultivation using GFP as a proxy. The non‐altered CEN.PK WT strain and CEN.PK pPr_Pho5_‐sfGFP were cultivated in starvation medium without trace elements and vitamins, only with inositol and zinc, as well as in P_i_‐rich feeding medium (Figure 6A). No increase in the OD‐corrected GFP signal for CEN.PK WT and CEN.PK pPr_Pho5_‐sfGFP in the P_i_‐rich medium was observed, validating the introduction of the plasmid to detect PHO pathway activation. In the absence of P_i_, the Pho5 promoter is induced by the PHO pathway. This is confirmed by a rising OD‐corrected GFP signal in experiments with CEN.PK pPr_Pho5_‐sfGFP. Regarding the medium composition, the increase was stronger in the medium with only zinc and inositol present than in the medium without trace elements and vitamins. The enhanced PHO pathway activation suggests an influence of either inositol or zinc or both on the starvation response. Parallel flask cultivations confirmed polyP hyperaccumulation in CEN.PK WT and CEN.PK pPr_Pho5_‐sfGFP (Figure S5).

Another cultivation containing only inositol or only zinc was performed to determine their contribution to PHO pathway activation (Figure 6B,C). This demonstrated that zinc is responsible for enhanced PHO pathway activation, as depicted by a nonsignificant difference in the rate of OD‐corrected GFP increase compared to the approach with zinc and inositol. The approach with inositol resulted in a nonsignificant difference compared to the approach without trace elements and vitamins. Therefore, it can be concluded that zinc plays a significant role in activating the PHO pathway under P_i_ starvation, thereby contributing to an enhanced subsequent polyP accumulation. The presence of extracellular inositol does not improve the starvation response via the PHO pathway.

## 4 Discussion

Prior research highlighted the addition of both trace elements and vitamins to the starvation medium to substantially increase polyP titer (Christ and Blank 2019). Here, we adapted previously published methods of media characterization to elucidate the beneficial components from either mixture, highlighting zinc and inositol as the major regulators (Figure 4) (Battling et al. 2020; Müller et al. 2018). Attributing the positive effect on polyP accumulation to inositol and zinc offers a wide range of opportunities for further optimization, including a deeper understanding of the polyP hyperaccumulation process, targeted process/enzyme engineering on inositol‐ and zinc‐utilization, strain‐specific media adaptation, and reduction of process cost by limiting trace element and vitamin usage in cultivations. Numerous cellular mechanisms are influenced by inositol and zinc, as both compounds play essential roles in general *S. cerevisiae* homeostasis. Zinc is required for the structural stability of proteins (Magonet et al. 1992), utilized among others as cofactor in transcription factors and alcohol dehydrogenases (Bird and Wilson 2020; Leskovac et al. 2002; Wilson and Bird 2016), or as signaling molecule (Regalla and Lyons 2006). These vital roles underline the involvement of zinc in a multitude of cellular processes, such as gene transcription (Vallee and Falchuk 1993), phospholipid metabolism (Henry et al. 2012), and stress response (Zhao and Bai 2012). 8.8% of the yeast genome is assumed to encode zinc‐binding proteins (Andreini et al. 2006). Therefore, it is challenging to pinpoint the exact genetic element or cellular mechanism for the observed polyP increase. A genome‐wide association study identified 108 single‐gene deletion mutants associated with zinc stress (6 mM), including *PHO85*, *PHO87, PHO90, VTC2, VTC4*, *VTC5, VMA5*, and *VMA16* (Zhao et al. 2020). Although the maximal zinc concentration in our hyperaccumulation method (0.08 mM) is distant from the concentrations tested by Zhao et al. (2020), a metabolic connection between zinc and polyP is still apparent. This is supported by other studies that report an influence of zinc on the activity of the endopolyphosphatase Ppn2p as well as on the activity and accumulation of the alkaline phosphatase Pho8p (Gerasimaitė and Mayer 2017; Kizawa et al. 2017). (Poly)phosphatases are essential to mobilize P_i_ and contribute to P_i_ homeostasis (Austin and Mayer 2020; Donella‐Deana et al. 1993). Although the connection to polyP hyperaccumulation remains uncharacterized, the PHO pathway plays a critical role. By using the Pho5‐regulated sensor for tracking PHO pathway activation during starvation, we observed a strong response to zinc addition. These results indicate that zinc directly contributes to P_i_ homeostasis and the starvation response. As a cofactor of transcription factors, it might stimulate the expression of PHO genes or influence the activity of proteins in the PHO pathway as a critical catalytic factor. As the promoter output of Pho5 is further downstream in the pathway, a large variety of additional regulations might influence the signal output (Chabert et al. 2023). Therefore, the exact mode of action and whether zinc acts directly or indirectly on one of the enzymes in the PHO pathway remains unclear and has not been investigated in this study. Recently, the role of inositol in the PHO pathway was elucidated by modulation with inositol pyrophosphates (InsPPs), ATP, and polyP on a metabolic and signaling level. On the one hand, this modulation plays a role in cellular P_i_ homeostasis, but on the other hand, it is also influenced by cellular P_i_ homeostasis itself (Azevedo and Saiardi 2017). The abundance of InsPPs changes depending on the cytosolic P_i_ level and thereby acts in conjunction with SPX (Syg1/Pho81/XPR1) domains to perform a coordinated P_i_ signaling response in *S. cerevisiae*. SPX domains are primarily found in proteins involved in P_i_ transport, P_i_ storage, and polyP synthesis (Secco et al. 2012; Wild et al. 2016). However, pinpointing the exact role of InsPPs in P_i_ signaling was hindered in the past due to analytical limitations, resulting in contradicting statements (Chabert et al. 2023; Gerasimaite et al. 2017). The study by Chabert et al. (2023) stressed the role of 1,5‐InsP8 in signaling cytosolic P_i_ levels, correlating P_i_ scarcity with an absence of 1,5‐InsP8 in *S. cerevisiae*. Our sensory system noted no increased starvation response via inositol addition. Hence, inositol may influence the starvation response independent of the PHO pathway by modulating phospholipid synthesis, transcription regulation, physiological stress response, energy metabolism, or autophagy (Henry et al. 2012; Lee et al. 2007; Shah et al. 2017; Shetty et al. 2013). The influence of inositol is limited exclusively to the starvation phase, as addition to the feed does not lead to increased polyP accumulation. When cultivating our strains under P_i_ starvation in TOM cultivations, increased fermentative activity was observed, which might be associated with InsPPs. Besides its role in P_i_ homeostasis, InsPPs function as metabolic signaling molecules, indicating cellular energy availability (Austin and Mayer 2020; Bennett et al. 2006; Szijgyarto et al. 2011). This sensing affects the carbon flux distribution, most prominently between fermentation and respiration. In this context, the enzymes controlling the reaction between InsP6 and 5‐InsP7 are of relevance. Qin et al. (2023) reported an inositol pyrophosphatase encoded by *OCA5* that dephosphorylates 5‐InsP7 to InsP6. Deletion of *OCA5* increased respiration and repressed fermentation. Szijgyarto et al. (2011) reported that deletion of *KCS1*, which catalyzes the reaction from InsP6 to 5‐InsP7, results in activation of glycolysis and repression of respiration. In both cases, the glucose uptake and ethanol production rates were affected (Qin et al. 2023; Szijgyarto et al. 2011). Although cultivations in this study were performed under P_i_ starvation conditions and without mutations concerning 5‐InsP7 and InsP6, increases in ethanol production and glucose uptake were observed via HPLC analysis. The added value of inositol during P_i_ scarcity might result from its role in maintaining P_i_ distribution between ATP, InsPP, and polyP. ATP is required by *S. cerevisiae*, but more respiration is impossible since fermentation occurs under the applied conditions due to the Crabtree effect (Pfeiffer and Morley 2014). Therefore, more carbon (glucose) is taken up by *S. cerevisiae* and converted to ethanol. In approaches with inositol, more ethanol is produced, which can lead to changes in P_i_ homeostasis through changes in energy homeostasis. Elevated ATP levels in those approaches can then produce more polyP in times of P_i_ repletion or lead to a stronger starvation response. It is striking that the polyP content in Ethanol Red does not change with varying inositol concentrations in the starvation phase compared to other tested strains (Figure 4). Ethanol Red produces high ethanol yields and maintains high cell viability under these conditions (Lesaffre 2025; Trivellin et al. 2022). Previous publications reported that elevated ethanol concentrations and tolerance in yeast strains are related to high levels of phosphatidylinositol in the cellular membrane with inositol supplementation (Chi et al. 1999; Furukawa et al. 2004). Ethanol tolerance and insensitivity to inositol supplementation in the starvation phase in Ethanol Red might hence be related to naturally high inositol production. This trait could result from domestication (Gallone et al. 2016). In summary, zinc has a positive effect via the direct activation of the PHO pathway during P_i_ starvation, while the role of inositol in enhanced polyP hyperaccumulation remains elusive. Since those two components are sufficient for enhancing polyP hyperaccumulation and even small concentrations triggered a boost in production, the starvation medium can be simplified by omitting unnecessary trace elements and vitamins. Next to the obvious ecological and economic advantages of saving resources, less usage of components can simplify product purification and, especially in this case, wastewater treatment, which is in turn beneficial for the footprint of the process.

## 5 Conclusion

Identifying zinc and inositol to enhance polyP hyperaccumulation opens new avenues to study P_i_ starvation in *S. cerevisiae*. The production of (bio‐)polyP is increasingly gaining industrial attention to replace energy‐intensive processes based on fossil resources. There is a great incentive for turning the yeast‐based approach into a profitable process. Applying our findings in combination with approaches in metabolic engineering and bioprocess development can support this cause. This study contributes to an improved understanding of polyP accumulation and a more efficient polyP production, thus to an envisaged circular P_i_ economy.

## Author Contributions

Alexander Deitert conceived the study and wrote the manuscript. Makarius Baier and Jana Fees constructed the strains applied in this study. Ailín Österlein Kück and Julia Repin performed most experiments. Roy Eerlings and Philipp Demling assisted in the manuscript curation. Lars M. Blank acquired funding, supervised the project, and revised the manuscript.

## Disclosure

Lars M. Blank filed two patent applications: “Zusammensetzung, enthaltend getrocknetes Polyphosphat und Verfahren zur Gewinnung von Polyphosphat aus polyphosphat‐haltigen Hefezellen dazu” (DE 10 2019 131 561.1) and “Polyphosphatreiche Hefeextrakte und Herstellverfahren dazu” (DE 10 2018 130 081.6, PCT/EP2019/082709).

## Conflicts of Interest

The authors declare no conflicts of interest.

## Supporting information


**Table S1:** Concentration of vitamin stock solutions. **Table S2:** Concentration of trace element stock solutions. **Figure S1:** Influence of individual trace elements of Group 3 on polyP hyperaccumulation of VH2.200. **Figure S2:** Influence of only inositol and only zinc on polyP hyperaccumulation of VH2.200. **Figure S3:** PolyP content of TOM cultivation with various yeast strains in starvation medium w/o any trace elements and vitamins (NE_TE+TV_) or only with inositol and ZnSO_4_. **Figure S4:** Plasmid map of pPr_Pho5_‐sfGFP to assay PHO pathway activation. **Figure S5:** PolyP content of BioLector cultivation with CEN.PK WT and CEN.PK pPr_Pho5_‐sfGFP.

## Data Availability

The data presented in this study are available on request from the corresponding author.
