# Supplementary material for: Polyphosphate Accumulation Is Determined by Zinc and Inositol in Saccharomyces cerevisiae
Source: Yeast. 2025 Oct 25;42(11):249–60. doi: 10.1002/yea.70006 (PMC12587025; doi:10.1002/yea.70006)
Supplement: Supplementary file 1 — Table S1: Concentration of vitamin stock solutions. Table S2: Concentration of trace element stock solutions. Figure S1: Influence of individual trace elements of Group 3 on polyP hyperaccumulation of VH2.200. Figure S2: Influence of only inositol and only zinc on polyP hyperaccumulation of VH2.200. Figure S3: PolyP content of TOM cultivation with various yeast strains in starvation medium w/o any trace elements and vitamins (NETE+TV) or only with inositol and ZnSO4. Figure S4: Plasmid map of pPrPho5‐sfGFP to assay PHO pathway activation. Figure S5: PolyP content of BioLector cultivation with CEN.PK WT and CEN.PK pPrPho5‐sfGFP. [file YEA-42-249-s001.docx]

# Supplementary

**Table S1. Concentration of vitamin stock solutions**

| **Vitamin** | **Medium concentration  [g/l]** | **Stock  concentration**  **[g/l]** | **Factor medium/stock concentration** | **pH**  **value** |
| --- | --- | --- | --- | --- |
| Biotin | 5.0 ⋅ 10^−5^ | 0.2 | 4000 | 6.5 |
| Nicotinic acid | 1.0 ⋅ 10^−3^ | 1.0 | 1000 | 6.5 |
| Calcium pantothenate | 1.0 ⋅ 10^−3^ | 1.0 | 1000 | 6.5 |
| p-aminobenzoic acid | 2.0 ⋅ 10^−4^ | 0.4 | 2000 | 6.5 |
| Pyridoxine HCl | 1.0 ⋅ 10^−3^ | 1.0 | 1000 | 6.5 |
| Thiamine HCl | 1.0 ⋅ 10^−3^ | 1.0 | 1000 | 6.5 |
| myo-Inositol | 2.5 ⋅ 10^−2^ | 25 | 1000 | 6.5 |

**Table S2. Concentration of trace element stock solutions**

| **Trace elements** | **Medium concentration  [g/l]** | **Stock  concentration**  **[g/l]** | **Factor medium/stock concentration** | **pH**  **value** |
| --- | --- | --- | --- | --- |
| Na_2_EDTA | 1.5 ⋅ 10^−2^ | 1.5 | 100 | 4.0 |
| ZnSO_4_ · 7 H_2_O | 4.5 ⋅ 10^−3^ | 0.45 | 100 | 4.0 |
| MnCl_2_· 2 H_2_O | 1.0 ⋅ 10^−3^ | 1 | 1000 | 4.0 |
| CoCl_2_ · 6 H_2_O | 0.3 ⋅ 10^−3^ | 0.3 | 1000 | 4.0 |
| CuSO_4_ · 5 H_2_O | 0.3 ⋅ 10^−3^ | 0.3 | 1000 | 4.0 |
| Na_2_MoO_4_ · 2 H_2_O | 0.4 ⋅ 10^−3^ | 0.4 | 1000 | 4.0 |
| KI | 0.1 ⋅ 10^−3^ | 0.2 | 2000 | 4.0 |
| CaCl_2_ · 2 H_2_O | 4.5 ⋅ 10^−3^ | 0.45 | 100 | 4.0 |
| FeSO_4_ · 7 H_2_O | 3.0 ⋅ 10^−3^ | 0.3 | 100 | 4.0 |
| H_3_BO_3_ | 1.0 ⋅ 10^−3^ | 0.2 | 200 | 4.0 |

**
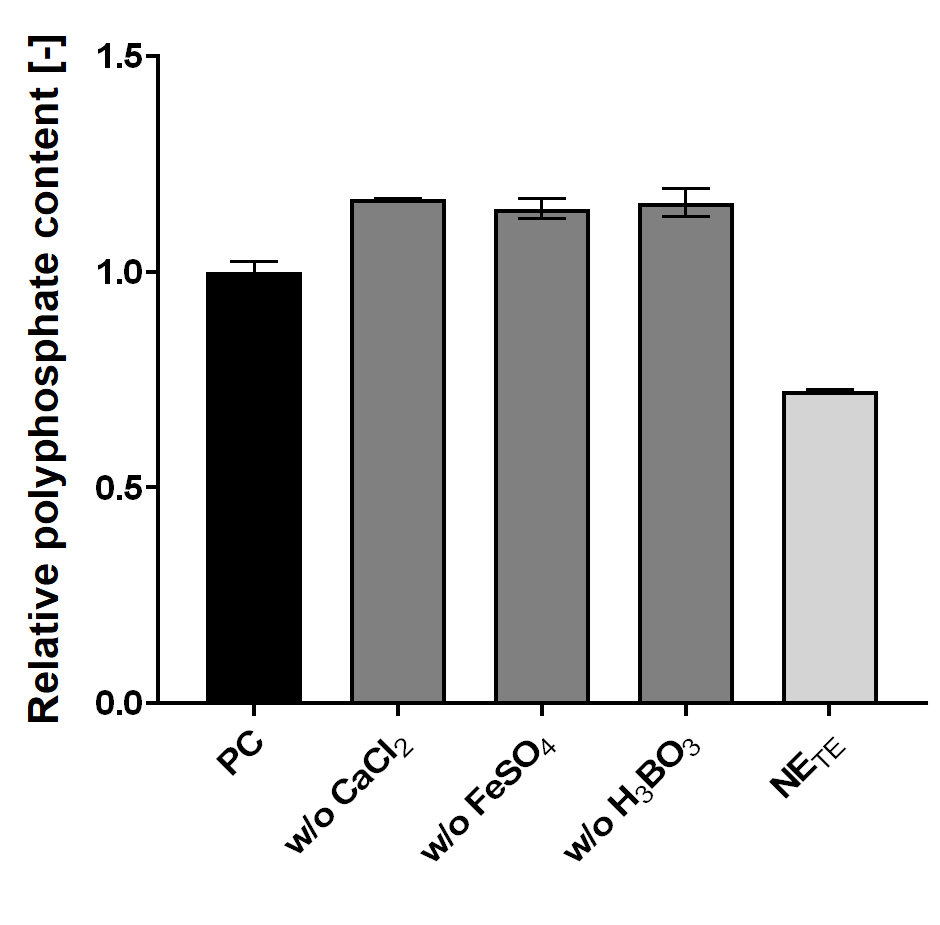
**

**Figure S1. Influence of individual trace elements of Group 3 on polyP hyperaccumulation of VH2.200**. PC (all trace elements), w/o CaCl_2_, w/o FeSO_4_, w/o H_3_BO_3,_ and NE_TE_ (w/o any trace elements). All cultivations were performed in duplicates (n = 2). Data are presented as means ± SEM.


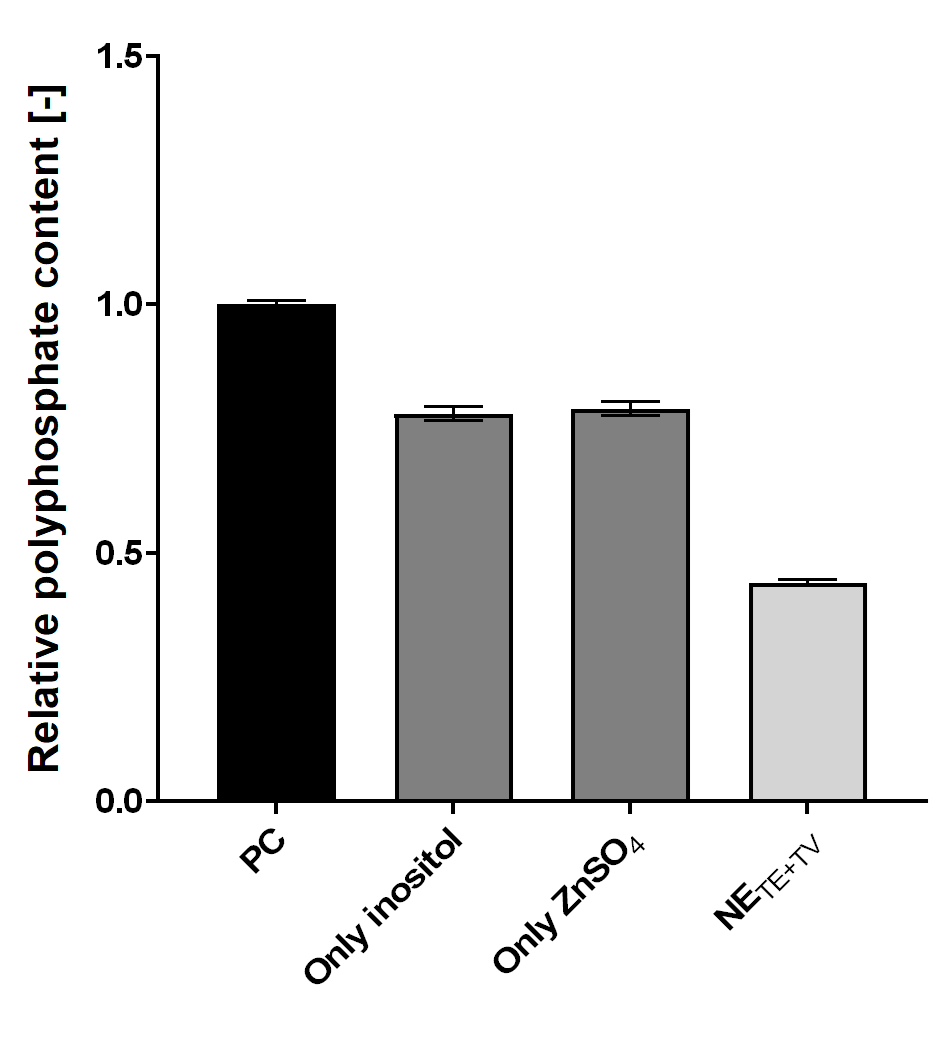


**FigureS2. Influence of only inositol and only zinc on polyP hyperaccumulation of VH2.200**. PC (all vitamins + trace elements), only with inositol, only with ZnSO_4_, and NE_TE+TV_ (w/o any trace elements and vitamins). All cultivations were performed in duplicates (n = 2). Data are presented as means ± SEM.


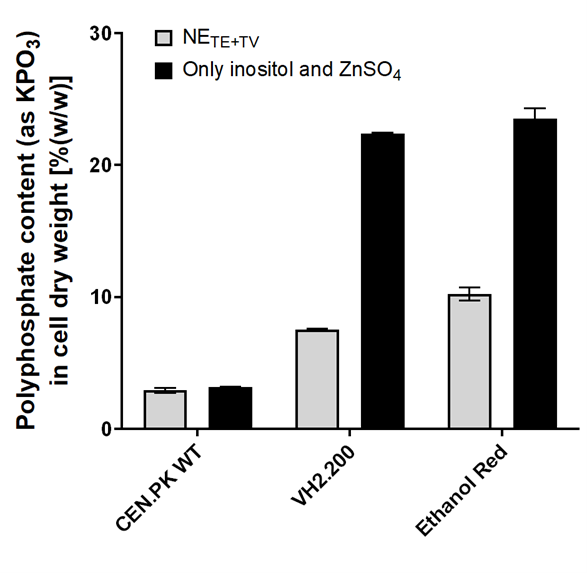


**Figure S3. PolyP content of TOM cultivation with various yeast strains in** **starvation medium w/o any trace elements and vitamins (NE_TE+TV_) or only with inositol and ZnSO_4_**. *S. cerevisiae* CEN.PK WT (125 mg/l inositol, 12.6 mg/l ZnSO_4_), VH2.200 (25 mg/l inositol, 2.5 mg/l ZnSO_4_), and Ethanol Red (25 mg/l inositol, 12.6 mg/l ZnSO_4_). All cultivations were performed in duplicates (n = 2). Data are presented as means ± SEM.


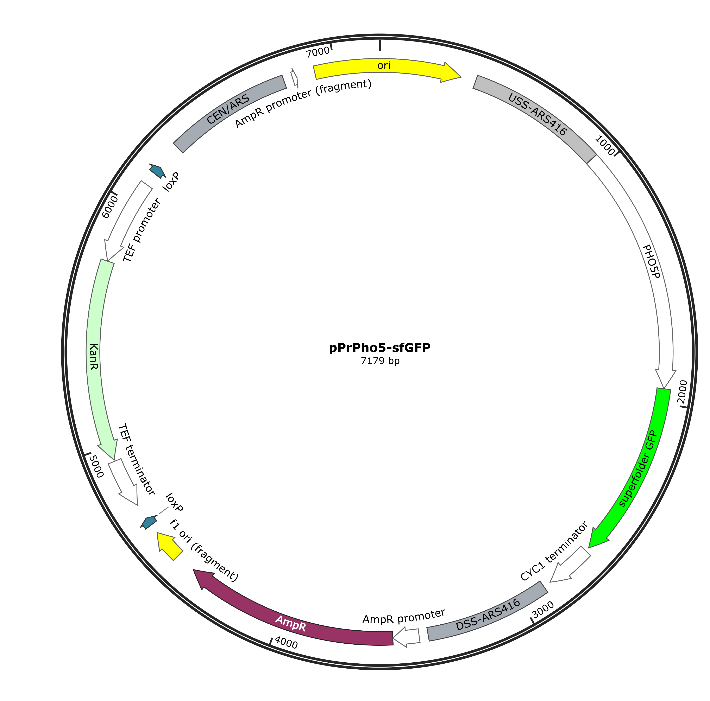


**Figure S4.** **Plasmid map of** **pPr_Pho5_-sfGFP to assay PHO pathway activation.** Centromeric plasmid carrying G418 resistance in CEN.PK 113-7D

**
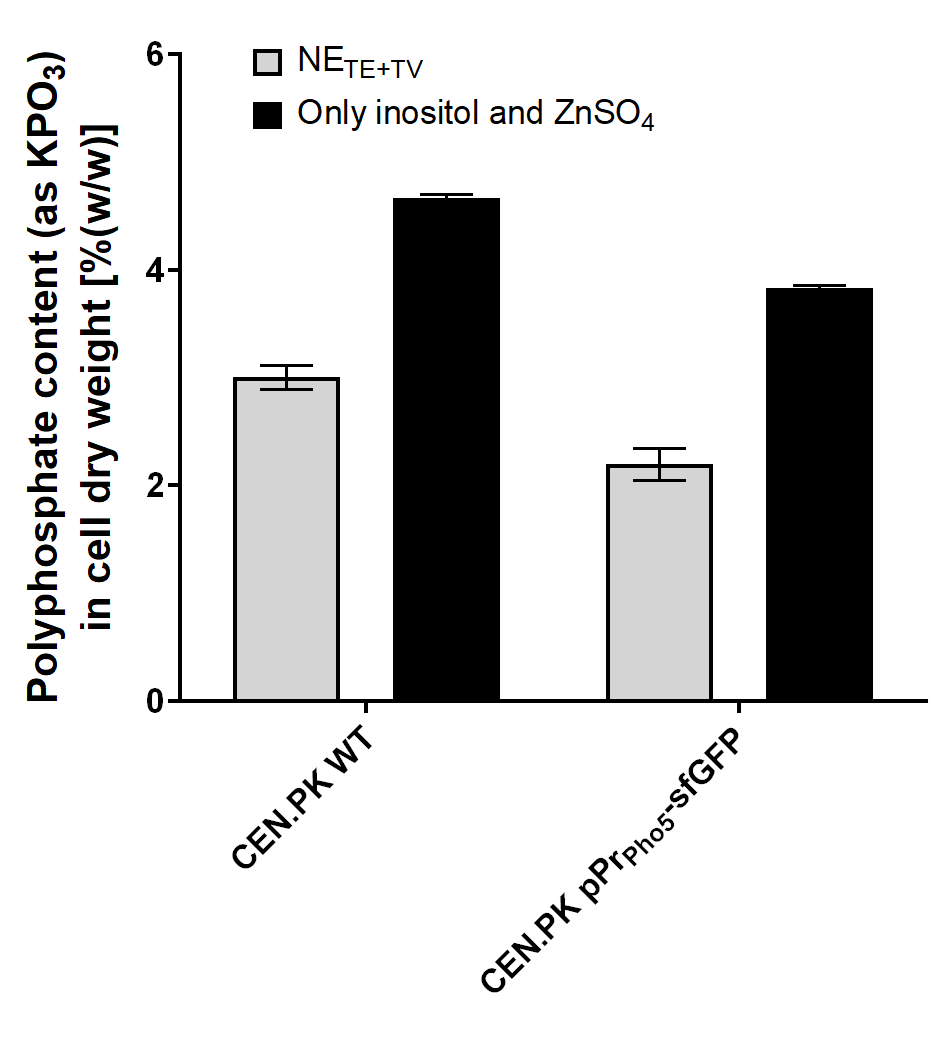
**

**Figure S5. PolyP content of BioLector cultivation with CEN.PK WT and CEN.PK pPr_Pho5_-sfGFP**. Starvation medium w/o any trace elements and vitamins (NE_TE+TV_) or only with inositol and ZnSO_4_. All cultivations were performed in duplicates (n = 2). Data are presented as means ± SEM.
